# Taste detection threshold of human (*Homo sapiens*) subjects and taste preference threshold of black-handed spider monkeys (*Ateles geoffroyi*) for the sugar substitute isomalt

**DOI:** 10.1007/s10329-020-00868-5

**Published:** 2020-10-03

**Authors:** Sofia Pereira, Laura Teresa Hernandez Salazar, Matthias Laska

**Affiliations:** 1 Department of Physics, Chemistry and Biology, Linköping University, 581 83 Linköping, Sweden; 2 Instituto de Neuro-Etologia, Universidad Veracruzana, 91000 Xalapa, Veracruz Mexico

**Keywords:** Taste detection threshold, Taste preference threshold, Isomalt, Human subjects, Spider monkeys, *Ateles geoffroyi*

## Abstract

The artificial sweetener isomalt is widely used due to its low caloric, non-diabetogenic and non-cariogenic properties. Although the sweetening potency of isomalt has been reported to be lower than that of sucrose, no data on the sensitivity of humans for this polyol are available. Using an up-down, two-alternative forced choice staircase procedure we therefore determined taste detection thresholds for isomalt in human subjects (*n* = 10; five females and five males) and compared them to taste preference thresholds, determined using a two-bottle preference test of short duration, in a highly frugivorous nonhuman primate, the spider monkey (*n* = 4; one female, three males). We found that both species detected concentrations of isomalt as low as 20 mM. Both humans and spider monkeys are less sensitive to isomalt than to sucrose, which is consistent with the notion of the former being a low-potency sweetener. The spider monkeys clearly preferred all suprathreshold concentrations tested over water, suggesting that, similar to humans, they perceive isomalt as having a purely sweet taste that is indistinguishable from that of sucrose. As isomalt, like most sweet-tasting polyols, may elicit gastric distress when consumed in large quantities, the present findings may contribute to the choice of appropriate amounts and concentrations of this sweetener when it is employed as a sugar substitute or food additive for human consumption. Similarly, the taste preference threshold values of spider monkeys for isomalt reported here may be useful for determining how much of it should be used when it is employed as a low-caloric sweetener for frugivorous primates kept on a vegetable-based diet, or when medication needs to be administered orally.

## Introduction

Isomalt is a widely used sugar substitute which provides only 2 kcal/g, i.e., half the energy value of sucrose (Radeloff and Beck 2013). It is a polyol comprising an equimolar mixture of two mutually diastereomeric disaccharides, each composed of two sugars: one of glucose and mannitol (1-*O*-α-d-glucopyranosyl-d-mannitol), the other of glucose and sorbitol (6-*O*-α-d-glucopyranosyl-d-sorbitol) (Evrendilek 2012). In contrast to conventional saccharides, isomalt is suitable for diabetics as no significant increase in body glucose, insulin or lactic acid concentration arises after its consumption (Thiébaud et al. 1984). Further, it is non-cariogenic, which makes isomalt attractive for use as a tooth-friendly candy (Featherstone 1994). Due to its low hygroscopicity and high chemical stability isomalt is particularly suitable for pharmaceutical applications, e.g., as a coating for tablets, to enhance the climate stability of pharmaceutical formulations containing moisture-sensitive active ingredients (Sentko and Bernard 2012). In contrast to a variety of other artificial sweeteners, isomalt has no bitter, metallic, or other unpleasant side tastes or aftertaste (Schiffman et al. 1995), but has been described as having a pure sweet taste indistinguishable from that of sucrose (Sentko and Willibald-Ettle 2012). Psychophysical studies have shown that isomalt is a low-potency sweetener perceived by humans as about 45–60% as sweet as sucrose, depending on the study and reference concentration used (DuBois et al. 1991). Surprisingly, no study so far has determined human taste detection thresholds for this artificial low-calorie sweetener.

Similar to human subjects, captive nonhuman primates face a variety of nutrition-related health issues such as obesity (Videan et al. 2007), diabetes (Kuhar et al. 2013), and caries (Crovella and Ardito 1994). This is particularly true for frugivorous primates as cultivated fruits, which often form the bulk of their captive diet, contain markedly higher carbohydrate concentrations compared to the fruits that they feed on in the wild (Schwitzer et al. 2009). Attempts to replace a fruit-based diet by a vegetable-based diet in captive frugivorous primates may alleviate the above-mentioned health issues (Schwitzer and Kaumanns 2003), but the diet needs to be balanced against the animals’ predilection for sweet-tasting fruits (Ramirez 1990) to prevent the refusal of less attractive food items. Here, a sugar substitute such as isomalt might be useful as a food complement or for orally administering medication due to its low-caloric, non-diabetogenic and non-cariogenic properties. However, no study has so far determined taste preference thresholds for this sugar substitute in any nonhuman primate species. It was therefore the aim of the present study to determine taste detection thresholds in human subjects and taste preference thresholds in a highly frugivorous primate species, the spider monkey.

Based on previous studies on sweet-taste perception in human and nonhuman primates, we hypothesized that (1) spider monkeys are able to perceive isomalt; (2) both human subjects and spider monkeys are less sensitive to isomalt than to sucrose; (3) spider monkeys are at least as sensitive to isomalt as human subjects; and (4) spider monkeys, similar to human subjects, perceive isomalt as purely sweet.

## Methods

### Human subjects

A total of ten healthy, unpaid volunteers (five males and five females) between 23 and 26 years of age participated in this study. They were all of Caucasian background, born and raised in western Europe, and not genetically related to each other. None of the subjects were smokers, had any history of olfactory or taste dysfunction, suffered from an acute upper respiratory or gastrointestinal tract infection, or were on any medication that might alter gustatory processing. None of the subjects had previously participated in studies on sweet-taste perception. They were informed as to the aim of the study and provided written consent for their participation in it. The study was performed in accordance with the Declaration of Helsinki, revised 2013.

### Animals

We assessed taste preference thresholds for isomalt in one adult female and three adult male black-handed spider monkeys *(Ateles geoffroyi)*. The animals were maintained at the field station Manejo para la Conservación de la Vida Silvestre (UMA) Doña Hilda Ávila de O’Farrill of the Universidad Vercruzana, near the town of Catemaco, in the province of Veracruz, Mexico. They were group housed in a series of roofed outdoor enclosures exposed to natural light and ambient temperatures that were connected to each other via sliding doors. The spider monkeys were between 8 and 12 years old at the start of the study and were not genetically related to each other. All of them had previously participated in studies on sweet-taste perception. We performed the tests in an empty enclosure as this allowed us to test the animals separately to avoid competition and distraction. All animals were trained to voluntarily enter the test enclosure and were completely accustomed to the procedure described below. The animals were fed fresh fruit and vegetables once per day. As spider monkeys do not normally drink from open water sources but meet their water requirements by consuming juicy fruits, they did not have access to water. Accordingly, no water deprivation schedule was adopted. The amount of food offered daily to the animals was such that leftovers were still present on the floor the next morning. Thus, it was unlikely that a ravenous appetite affected the animals’ ingestive behavior. Testing took place in the morning, prior to feeding.

The experiments reported here comply with the American Society of Primatologists’ Principles for the Ethical Treatment of Primates, and also with current Swedish and Mexican laws. The study was performed according to a protocol approved by the Ethical Board of the Federal Government of Mexico’s Secretariat of Environmental and Natural Resources (official permits no. 09/GS-2132/05/10).

### Taste stimuli

Isomalt (CAS no. 64519-82-0) of the highest available purity (< 99.5%) was used. It was obtained from Beneo-Palatinit (Mannheim, Germany).

### Human test procedure

We used an up-down, two-alternative forced choice staircase procedure to determine taste detection thresholds (Snyder et al. 2015). Each subject was asked to indicate which of two simultaneously presented liquids (5 ml each) contained the taste stimulus and which one contained tap water only. Each subject was allowed to swallow the sampled liquid and was asked to rinse his/her mouth with water between each stimulus. Testing started at a clearly detectable concentration of isomalt (160 mM) which was then decreased in twofold concentration steps until the subject failed to detect the substance. Each concentration was presented twice to the subject and two correct choices resulted in a subsequent decrease in concentration in the following set of two trials. An incorrect choice, i.e., failing to correctly identify the liquid containing the taste stimulus, was followed by an increase in concentration. Testing terminated once a subject reached seven reversals, i.e., seven turning points in the direction of the concentration staircase, and the median value of the concentrations one dilution step above the reversals was taken as the subject’s detection threshold value. All subjects were tested at least 1 h after they had completed a meal.

### Spider monkey test procedure

We used a two-bottle preference test of short duration (Richter and Campbell 1940). The animals were allowed to drink for 1 min from a pair of simultaneously presented graduated cylinders with metal drinking spouts. We performed three of such 1-min trials per day and animal, with intertrial intervals of approximately 30 min. Testing took place in the morning, prior to feeding the animals their daily ration of food. To determine taste preference thresholds the animals were given the choice between tap water and defined concentrations of isomalt dissolved in tap water. Testing started at a concentration of 500 mM and proceeded at concentrations of 100, 50, 20, 10, and 5 mM until an animal failed to show a significant preference. We presented each pair of stimuli ten times per individual animal, and the position of the stimuli was pseudo-randomized in order to counterbalance possible position preferences. Care was taken that an animal sampled both stimuli at least once during each trial. To maintain the animals’ motivation and willingness to cooperate, testing of the different stimulus concentrations did not follow a strictly descending or ascending order but was pseudo-randomized. This was true both within the three trials performed on a given testing day and between days.

For each animal, we recorded the amount of liquid consumed from each bottle, summed it for the ten trials with a given stimulus combination, converted it to a percentage (relative to the total amount of liquid consumed from both bottles), and took 66.7% (i.e., 2/3 of the total amount of liquid consumed) as the criterion of preference. We chose this rather conservative criterion for reasons of comparability of data, as the same criterion had been used in previous studies on sweet-taste responsiveness with both spider monkeys (Laska et al. 1996, 1998, 1999a, 2001) and other primate species (Laska et al. 1999b; Laska 2000; Wielbass et al. 2015; Nicklasson et al. 2018; Norlén et al. 2019), and in order to avoid misinterpretation due to a too liberal criterion. Additionally, we performed binomial tests and regarded an animal as significantly preferring one of the two stimuli if it reached the criterion of 66.7% and consumed more from the bottle containing the preferred stimulus in at least eight out of ten trials (binomial test, *p* < 0.05). Thus, we defined taste preference threshold as the lowest concentration at which the animals met both criteria mentioned above.

Preliminary analyses of the data indicated that there were no systematic differences in choice behavior and liquid consumption between the three 1-min trials performed on a given day. Intraindividual variability of the amount of liquid consumed across the ten trials with a given stimulus combination was low and averaged less than 20%. Thus, a theoretically possible bias in the overall preference score due to excessive drinking in aberrant trials did not occur.

## Results

### Human subjects

**Fig. 1:**
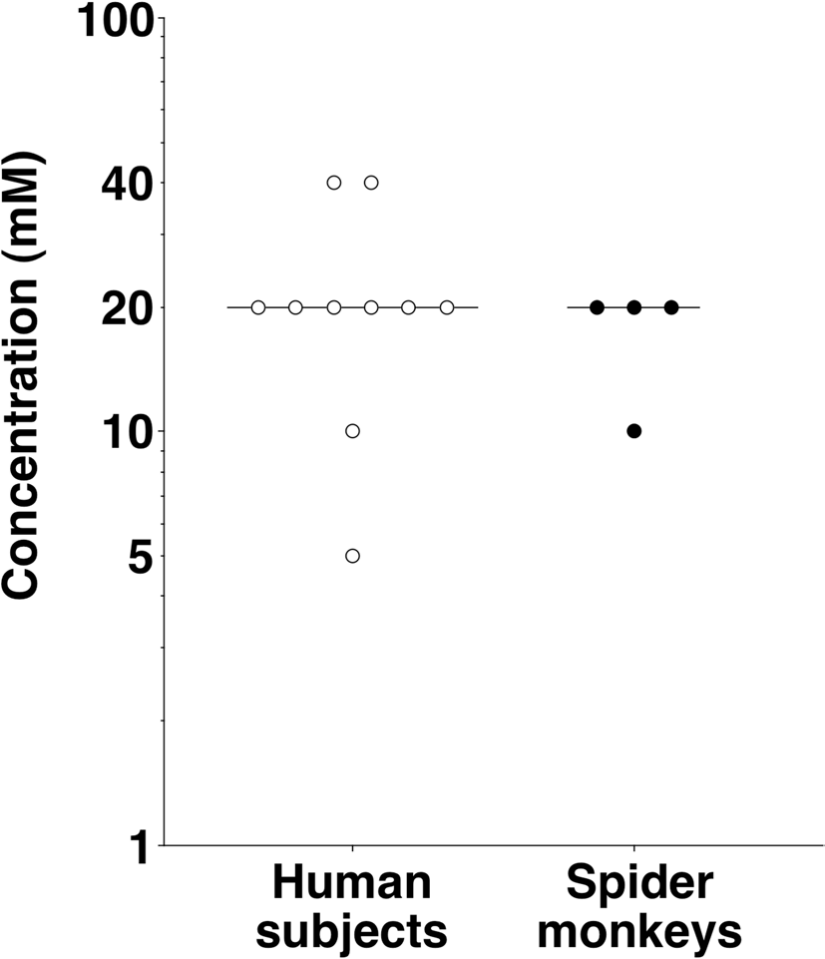
Taste* detection* thresholds of ten human subjects (*white circles*) and taste* preference* thresholds of four spider monkeys (*black circles*) for isomalt.* Each data point* represents the threshold value of one individual. The* horizontal lines* indicate the median threshold value for each of the two species

Taste detection thresholds of the ten human subjects for isomalt ranged from 5 to 40 mM (Fig. 1), with a median value of 20 mM. Male and female subjects did not significantly differ in their sensitivity for isomalt (Mann–Whitney *U*-test, *p* > 0.05).

### Spider monkeys

**Fig. 2:**
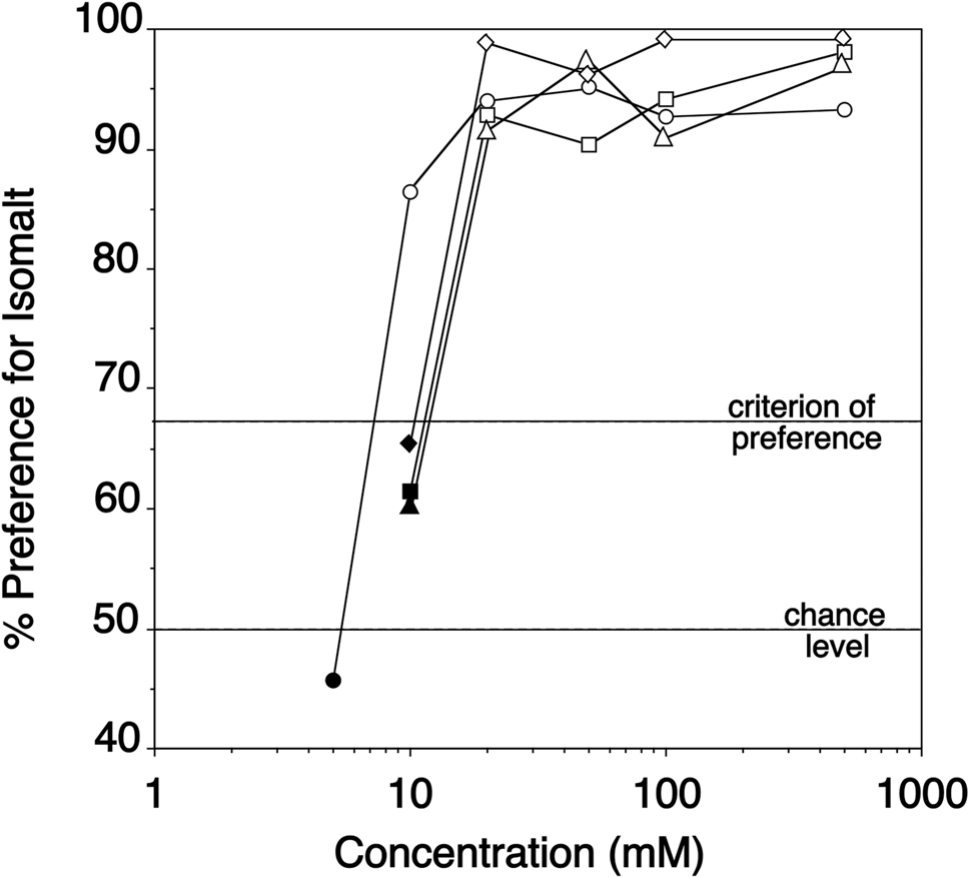
Taste responses of four spider monkeys to aqueous solutions of isomalt tested against tap water. *Each data point* represents the mean value of ten 1-min trials per animal. The four different symbols represent data for the four individual animals.* White symbols* indicate concentrations for which the criterion of preference was met, and* black symbols* indicate concentrations for which this was not the case. The* horizontal lines* at 66.7% and at 50% indicate the criterion of preference and the chance level, respectively

Taste preference thresholds of the four spider monkeys for isomalt ranged from 10 to 20 mM (Fig. 1), with a median value of 20 mM. (A threshold value of 10 mM isomalt was only reached in the test with the female.) All four animals failed to show a significant preference for the lowest concentrations presented, suggesting that the preference for higher concentrations of isomalt was indeed based on the chemical nature, i.e., the sweetness, of the stimulus. The animals did not display any rejection responses or a decrease in the degree of their preference for any of the suprathreshold concentrations tested. Rather, all suprathreshold concentrations of isomalt between 20 and 500 mM were preferred at more than 90% (Fig. 2).

## Discussion

The results of the present study demonstrate that both human subjects and spider monkeys display a taste threshold of 20 mM for isomalt. However, when comparing gustatory sensitivity between these two species one should take into account the fact that the taste *detection* threshold of the human subjects was established using a signal detection procedure, whereas the taste *preference* threshold of the spider monkeys only provides a conservative approximation of an animal’s taste sensitivity (Spector 2003). Thus, it seems reasonable to assume that spider monkeys are at least as sensitive, and possibly even more sensitive than human subjects for this artificial sweetener, which is consistent with our third hypothesis. This notion is in line with previous studies that reported spider monkeys to be more sensitive than humans for other sweet-tasting substances such as sucrose, fructose, glucose, and maltose, possibly due to their highly frugivorous diet (Laska et al. 1996). Although our sample size (*n* = 4) of spider monkeys was relatively small, the results appear robust as interindividual variability among the genetically non-related animals was low with a dilution factor of only 2 between the threshold values of the most and the least sensitive animal.

Our finding that the human taste detection threshold of 20 mM for isomalt was higher than the corresponding threshold value of 5.5 mM reported for sucrose (Low et al. 2017) is in agreement with other studies that found the sweetening potency of isomalt as perceived by humans to be lower than that of sucrose (DuBois et al. 1991). Similarly, the taste preference threshold of the spider monkeys of 20 mM for isomalt was higher than the corresponding threshold value of 3 mM for sucrose (Laska et al. 1996). Although we did not systematically assess the relative sweetness of isomalt in the spider monkeys, occasional tests in which we presented the animals with equimolar suprathreshold concentrations of both sucrose and isomalt clearly indicated that they preferred the former over the latter. This suggests that spider monkeys, too, perceive isomalt as less sweet compared to sucrose, and thus that this sugar substitute can be considered as a low-potency sweetener in this nonhuman primate species as well, which is consistent with our second hypothesis.

Our finding that the spider monkeys were clearly able to perceive isomalt, which is consistent with our first hypothesis, is not trivial considering that other artificial sweeteners such as aspartame, neotame, cyclamate,* N*-α-l-aspartyl-(*R*)-α-methylphenethylamine and* N*-α-l-aspartyl-l-(*O*-*tert*-butyl)serine methyl ester were found to be perceptible only for catarrhine primates, but not for platyrrhine primates such as the spider monkey (Glaser et al. 1992, 1996; Nofre et al. 1996). One possible explanation for the detection of isomalt by both catarrhine and platyrrhine primates is that, although the substance itself is not found in nature, its constituents (glucose, sorbitol, and mannitol) are found in a variety of fruits and other parts of plants consumed by primates. Accordingly, it seems reasonable to assume that the primate sweet-taste receptor coevolved with these sweet-tasting constituents of isomalt, and may thus have gained the ability to also interact with the resulting disaccharides as ligands.

The fact that the spider monkeys displayed a marked preference for isomalt even at the highest concentration tested (500 mM) suggests that, similar to human subjects, they perceive isomalt as purely sweet with no unpleasant bitter or metallic side taste or aftertaste (Sentko and Willibald-Ettle 2012), which is consistent with our fourth hypothesis. This, too, is not trivial considering that a variety of artificial sweeteners such as aspartame and sodium cyclamate have been reported to elicit an unpleasant side taste or aftertaste in humans when presented at high concentrations (Schiffman et al. 1995), and considering that spider monkeys, too, have been reported to reject high concentrations of these tastants, possibly due to an unpleasant taste (Pereira 2020).

Isomalt has been approved as a food additive for human consumption both in the European Union (E953) and in the United States. Nevertheless, like most sweet-tasting polyols, isomalt carries the risk of causing gastric distress, including flatulence and diarrhea, when consumed in large quantities (Evrendilek 2012). The present findings concerning the taste sensitivity of humans for isomalt may therefore contribute to the choice of appropriate amounts and concentrations when it is employed as a sugar substitute or food additive. Similarly, the taste preference threshold values of spider monkeys for isomalt reported here may be useful when isomalt is employed as a low-caloric sweetener for frugivorous primates kept on a vegetable-based diet or as a coating for tablets when medication needs to be administered orally.
